# Building a program of research in medical education: recommendations from the professional literature

**DOI:** 10.1080/10872981.2025.2546869

**Published:** 2025-08-12

**Authors:** Rebekah Cole

**Affiliations:** a Department of Military and Emergency Medicine, Uniformed Services University of the Health Sciences, Bethesda, MD, USA; b Department of Health Professions Education, Uniformed Services Unversity of the Health Sciences, Bethesda, MD, USA

**Keywords:** Medical education research, program of research, research productivity, professional identity, scholarly identity, faculty development, academic scholarship

## Abstract

Medical educators are increasingly expected to engage in impactful scholarship. However, many struggle to progress beyond isolated studies toward developing cohesive, sustainable programs of research that establish scholarly identity and contribute meaningfully to the field. Although literature exists on faculty development and individual research projects, comprehensive guidance for building a unified research trajectory in medical education remains limited. This narrative review aimed to identify and synthesize recommendations from the professional literature on developing a program of research in medical education. Searches of PubMed, MEDLINE, and Web of Science for English-language publications from January 2005 to April 2024 used terms such as ‘medical education,’ ‘program of research,’ and ‘research productivity.’ Twenty-five articles meeting inclusion criteria – including empirical studies, reviews, and perspectives offering practical guidance – were reviewed and analyzed thematically. Eight key strategies emerged for building a research program: (1) defining a focused program of research around core questions; (2) aligning research with institutional priorities to leverage support and resources; (3) grounding inquiries in theoretical and conceptual frameworks to enhance rigor; (4) transitioning from isolated studies to a cohesive research agenda; (5) identifying and securing resources, including mentorship and funding; (6) disseminating work strategically to maximize impact; (7) cultivating mentorship and collaborative networks; and (8) sustaining momentum while mitigating burnout risks. Despite these insights, significant gaps remain, including the lack of empirically tested frameworks specific to building programs of research in medical education and limited understanding of how these strategies apply across diverse institutional or cultural contexts. Developing a program of research in medical education requires deliberate focus, strategic alignment, and collaborative engagement. The synthesized recommendations offer practical pathways for educators aiming to enhance scholarly identity and productivity. Future research should explore the implementation and effectiveness of these strategies across varied settings to advance faculty development and the field’s scholarly impact.

## Introduction

The expectation for medical educators to engage in rigorous, impactful scholarship has grown significantly over the past two decades [1,2]. However, many educators may struggle to move beyond conducting isolated studies toward building a cohesive program of research that establishes scholarly identity and contributes lasting insights to the field [3]. Developing a program of research helps establish scholarly identity and contributes meaningfully to theoretical and practical knowledge in a field.

While there is literature on career development and conducting individual research projects within medical education [1,4–6], there remains a need for more guidance on how to transition from isolated studies to cohesive programs of research. To close this knowledge gap, this narrative review draws on recent medical education professional literature to highlight strategies that can support scholars in building a cohesive and sustainable program of research, and to identify gaps where future research is needed.

## Materials and methods

The purpose of this narrative review was to identify and synthesize the professional literature offering guidance and recommendations for building a program of research in medical education. A search strategy was designed to capture publications relevant to research career development, scholarly identity, faculty development, and practical strategies for sustaining a program of research. Searches were performed in the databases PubMed, MEDLINE, and Web of Science for English-language articles published between January 2005 and April 2024. Search terms included combinations of the following keywords: ‘medical education,’ ‘faculty development,’ ‘program of research,’ ‘academic scholarship,’ ‘career development,’ ‘early career development,’ ‘research strategy,’ and ‘research productivity.’ Reference lists of selected articles were also reviewed to identify additional relevant publications.

Inclusion criteria were:

- Articles focused on health professions education
- Articles offering recommendations, frameworks, or practical guidance relevant to developing a program of research within health professions education
- Narrative reviews, perspective pieces, systematic reviews, and empirical studies that included discussion of increasing scholarly activity and/or building a research agenda within health professions education

Exclusion criteria were:

- Articles published in a language other than English

The titles and abstracts of each article were reviewed to determine their relevance. After that, the articles were read in detail to determine themes related to building a program of research in medical education. The findings were grouped by theme, and important references from the literature are presented in the Results and Discussion sections to help explain and verify the recommendations in this review.

## Results

This narrative review included a total of 25 articles published between 2004 and 2024. The literature comprised empirical research studies (*n* = 17), narrative or integrative reviews (*n* = 7), and perspective or commentary pieces (*n* = 1). While many articles addressed faculty development, scholarly identity, or academic career advancement in medical education, relatively few focused explicitly on building a cohesive program of research. Nonetheless, recurring themes and practical recommendations emerged across the literature, reflecting a convergence of scholarly guidance for developing and sustaining a program of research in the health professions education context.

## Synthesized recommendations from the literature

### Define a focused program of research

Defining a focused program of research is the first step for researchers aiming to build a coherent and impactful body of work in medical education. A program of research represents an evolving set of investigations centered around a core problem or persistent set of questions, enabling scholars to establish a clear identity, and make substantial contributions to the field [1,5]. The professional literature emphasizes that clarity of focus strengthens both scholarly credibility and the potential for broader impact [3]. To develop such focus, emerging researchers are encouraged to reflect deeply on recurring challenges they observe in their teaching or clinical practice, to identify significant gaps that remain unexplored in the existing literature, and to consider personal passions that could sustain their intellectual curiosity and engagement over the long term [3,7]. By approaching the development of a program of research aligned to both personal interests and disciplinary needs, scholars are better positioned to produce work over an extended amount of time that is relevant and in-demand within their field [5,8]. Finally, defining a focused program of research also positions scholars for career advancement, as research productivity is strongly associated with academic rank and professional recognition in medicine [9].

### Align research with institutional priorities and expertise

Programs of research that align closely with institutional missions often secure stronger support and long-term sustainability, as institutions are more likely to invest in scholarship that advances their strategic goals and values [1,5]. When educators intentionally align their research interests with institutional priorities, they may be better positioned to access resources, gain administrative support, and create networks to enhance their work [4,10]. For example, educational researchers might explore how their interests intersect with advancing competency-based education through innovative AI, or developing innovative assessment strategies that improve learning outcomes and program effectiveness [11]. By seeking alignment between personal scholarly passions and organizational priorities, researchers not only find greater personal fulfillment, but also enhance the likelihood of institutional backing and broader scholarly impact [3,12].

### Ground inquiry in theoretical and conceptual frameworks

Contemporary literature underscores the crucial role of grounding medical education research in well-defined theoretical and conceptual frameworks, as these foundations enhance both the rigor and interpretability of scholarly work within medical education [3,7]. Theoretical frameworks are foremost essential foundations for research design as they offer broad explanations for how and why certain educational phenomena occur, providing scholars with lenses through which to understand complex processes. For example, Social Learning Theory helps explain how learners acquire behaviors through observation and modeling [13]. Similarly, conceptual frameworks are essential for clarifying the relationships among key elements in a study. For example, the Kirkpatrick Model is often used as a framework to evaluate the effectiveness of educational training programs by examining their strengths and weaknesses [14]. Educational paradigms, finally, encompass the underlying philosophical beliefs about how knowledge is constructed and how learning occurs [15]. For example, constructivism describes how students create their own meaning making, and educators can act like facilitators of this meaning making process. Ultimately, researchers who explicitly articulate their chosen frameworks and paradigms not only strengthen the coherence of their research, but may also increase chances of publication [7].

### Transition from isolated projects to a cohesive agenda

In order to build a strong program of research, scholars might approach each research project as a ‘puzzle piece’ of a broader scholarly narrative, rather than treating individual studies as isolated efforts [1,5]. For instance, a research study about feedback within a certain course might serve as a foundational pilot study that will eventually involve multi-institutional collaborations, resulting in a greater impact on the field [12,16]. In addition, specialty-focused research programs, such as those in orthopaedic sports medicine and foot and ankle surgery, demonstrate how initially isolated studies can evolve into cohesive research agendas contributing to broader scholarly impact [17,18]. Engaging colleagues across disciplines and institutions not only brings diverse perspectives and methodological strengths to the research process, but also extends the reach and influence of scholarly work [5,10]. Networking at conferences and through professional organizations is essential to building these collaborative relationships that lead to greater scholarly output [4,19].

### Identify and secure resources

Building a sustainable program of research in medical education demands investments of time, funding, mentorship, and institutional support [1,10]. It may be helpful for early career faculty to pursue small, internal grants, which not only provide initial funding but also help develop grant-writing skills and establish a record of scholarly productivity that can support larger grant applications in the future [4,5]. Collaborating with experienced researchers, especially when seeking large-scale external funding, can be beneficial for learning how to write competitive proposals [16,19,20]. When applying for intramural funding, clearly articulating how research aligns with departmental or institutional goals may increase the likelihood of administrative support and resource allocation [21,22].

### Disseminate strategically

To develop a program of research, recent literature emphasizes the importance of adopting a strategic approach to dissemination, beginning with carefully selecting journals that align with a researcher’s methodological approaches and theoretical frameworks [1]. Strategically selecting publication outlets is crucial, as journal placement and publication productivity often influence academic progression and visibility across medical specialties [23,24]. Additionally, presenting research at conferences in the form of workshops, abstracts, and oral presentations provides valuable opportunities for sharing findings and making key professional contacts that can foster future collaborations [5,16,19]. Leveraging social media platforms such as LinkedIn may also help to connect with diverse audiences and disseminate ideas widely and rapidly [25].

### Cultivate mentorship and collaborative networks

Meaningful professional relationships are essential to building a sustained program of research. The medical education literature underscores mentorship and networking as fundamental cornerstones of academic growth and success [1,5,6]. Educational researchers might consider identifying mentors who can offer strategic guidance, constructive feedback, and support for navigating the complexities of publishing within academic medicine [19]. In addition to mentorship, seeking out sponsors who can provide crucial access to leadership roles, funding opportunities, and influential professional networks is critically important for research development and advancement [10,16]. Finally, engaging in reciprocal mentorship by supporting and collaborating with junior colleagues not only contributes to their growth, but also can lead to expanded networks, fresh energy and perspectives, and increased scholarly output through collaborative projects, publications, and presentations [12].

### Sustain momentum and prevent burnout

Long-term scholarly productivity in medical education depends on maintaining a long-term balance between professional engagement and personal well-being [26]. Strategies to support this balance include setting realistic milestones and pacing projects to avoid overcommitment and burnout [12,19]. Researchers might seek out professional coaching as well in order to gain support and perspective in problem solving and career growth [27]. Actively participating in academic communities can also provide researchers with fresh perspectives and a sense of belonging, all of which can help sustain positive energy and perspective for research and teaching [1,10]. Ultimately, maintaining a healthy balance between scholarly pursuits and personal life has been found to be essential for long-term career success and fulfillment [16].

## Gaps identified in the professional literature

While the professional literature offers some practical recommendations for building a program of research, several gaps remain. No publications provide comprehensive, empirically tested frameworks specifically designed for medical education researchers seeking to build a program of research. Additionally, there is no research on how these strategies translate across diverse institutional settings, resource levels, or cultural contexts. Given this gap, future studies exploring the effectiveness of faculty development initiatives related to sustained scholarly productivity of various modalities are therefore warranted.

## Discussion

This narrative review identified key recommendations from the literature to guide medical educators in building a sustainable and impactful research program of research. Across the 25 health professions journal articles reviewed, themes emerged emphasizing the importance of focusing research on a cohesive set of questions, aligning scholarly activities with institutional priorities, grounding studies in theoretical and conceptual frameworks, and engaging in collaborative networks. These strategies collectively contribute to the development of a strong scholarly identity and long-term research success.

The findings of this review highlight practical pathways for medical educators seeking to transition from conducting isolated research projects to creating a coherent program of research. For early- and mid-career faculty, adopting these strategies may not only enhance scholarly productivity, but also increase opportunities for professional growth and leadership within the medical education community. Institutions, in turn, can design professional development training that supports sustained scholarly engagement in order to maximize the growth and development of medical education faculty, as well as scholarly output that optimizes medical education and training.

While prior literature has explored aspects of research career development in various fields, this review expands on existing work by synthesizing practical recommendations specifically tailored to the context of medical education research. However, there are several limitations to this review for consideration. As a narrative review, the findings may reflect selection bias. Additionally, the review focused on English-language literature, potentially limiting the inclusion of perspectives from non-English-speaking contexts. Overall, there was a limited number of studies that specifically focused on building a program of research within medical education to draw from in these recommended best practices.

Given these limitations, future research might fill this gap by exploring how these recommended strategies impact the scholarly productivity of educational researchers both short and long term, and explore the effectiveness of specific faculty development interventions aimed at promoting research career success. Studies investigating the challenges to implementing these recommendations, especially those faced by educators in diverse cultural and resource environments, may also be warranted.

## Conclusion

Developing a program of research in medical education is a complex, iterative endeavor grounded in both strategic planning and scholarly curiosity. This narrative review consolidates literature-informed recommendations to guide educators in cultivating impactful, sustainable research programs. As the field continues to evolve, educators who approach research intentionally and collaboratively can play a pivotal role in shaping the future of medical education scholarship.

## References

[1] Gruppen LD. The department of medical education at the University of Michigan medical school: a case study in medical education research productivity. Academic Med. 2004;79(10):997–6. doi: 10.1097/00001888-200410000-00023 15383363

[2] Ten Cate O. Health professions education scholarship: the emergence, current status, and future of a discipline in its own right. FASEB Bioadv. 2021;3(7):510–522. doi: 10.1096/fba.2021-00011 34258520 PMC8255850

[3] Holzemer WL. Building a program of research. Jpn J Nurs Sci. 2009;6(1):1–5. doi: 10.1111/j.1742-7924.2009.00115.x 19566633

[4] El-Sawi NI, Sharp GF, Gruppen LD. A small grants program improves medical education research productivity. Academic Med. 2009;84(10 Suppl):S105–S108. doi: 10.1097/ACM.0b013e3181b3707d 19907368

[5] Perry M, Hopson L, House J, et al. Model for developing educational research productivity: the medical education research group. West J Emerg Med. 2015;16(6):947–951. doi: 10.5811/westjem.2015.9.27306 26594297 PMC4651601

[6] Laupland KB, Edwards F, Dhanani J. Determinants of research productivity during postgraduate medical education: a structured review. BMC Med Educ. 2021;21(1), Article 567. doi: 10.1186/s12909-021-03010-1 PMC8579624 34753470

[7] Finnell DS. Building a program of research based on the transtheoretical model. J Addict Nurs. 2005;16(1–2):13–21. doi: 10.1080/10884600590917156

[8] Fortney CA. Palliative and end-of-life care for infants and their families in the NICU: building a program of research. J Pediatr Nurs. 2019;49:104–105. doi: 10.1016/j.pedn.2019.09.019 31668673 PMC6942219

[9] Zaorsky NG, O’Brien E, Mardini J, et al. Publication productivity and academic rank in medicine: a systematic review and meta-analysis. Academic Med. 2020;95(8):1274–1282. doi: 10.1097/ACM.0000000000003185 32028299

[10] Coppola KM, Rees Willett L, Galt J, et al. Increasing faculty productivity through an interprofessional research group. Med Educ. 2020;54(11):1057–1058. doi: 10.1111/medu.14324 32885483

[11] Crabtree BF, Nutting PA, Miller WL, et al. Primary care practice transformation is hard work: insights from a 15-year developmental program of research. Med Care. 2011;49(12 Suppl):S28–S35. doi: 10.1097/MLR.0b013e3181cad65c 20856145 PMC3043156

[12] Bednarek R, Cacciatori E, Chalkias K, et al. Delivering impact via the ebb-and-flow of a research team: reflection on a long-term program of research into a global societal challenge. J Appl Behav Sci. 2024;60(1):194–214. doi: 10.1177/00218863231207873

[13] Schunk DH, DiBenedetto MK. Albert Bandura’s legacy in education. Theory Into Pract. 2023;62(3):205–206. doi: 10.1080/00405841.2023.2226560

[14] Rouse DN. Employing Kirkpatrick’s evaluation framework to determine the effectiveness of health information management courses and programs. Perspect Health Inf Manag/. 2011;8(Spring):1c. PMC3070232 21464860

[15] Baker LR, Phelan S, Woods NN, et al. Re-envisioning paradigms of education: towards awareness, alignment, and pluralism. Adv Health Sci Educ Theory Pract. 2021;26(3):1045–1058. doi: 10.1007/s10459-021-10036-z 33742339 PMC8338841

[16] Peterson W, Santen SA, House JB, et al. Increasing education research productivity: a network analysis. West J Emerg Med. 2019;21(1):163–168. doi: 10.5811/westjem.2019.12.44512 31913839 PMC6948696

[17] Clark SC, Sanborn L, Brown SM, et al. Research productivity of orthopaedic sports medicine fellowship programs in the United States. Arthrosc, Sports Med Rehabil. 2021;3(4):e997–e1002. doi: 10.1016/j.asmr.2021.02.007 34430878 PMC8365224

[18] Khwaja AM, Bridge N, Sherman N, et al. Foot and ankle fellowship faculty research productivity. Foot Ankle Orthop. 2020;5(4), Article 2473011420961023. doi: 10.1177/2473011420S00287 33158371

[19] Bragg M, Arshonsky J, Pageot Y, et al. Student-led research team-building program may help junior faculty increase productivity in competitive biomedical research environment. BMC Med Educ. 2021;21(1), Article 3. doi: 10.1186/s12909-020-02396-8 PMC7784259 33397349

[20] Morales DX, Grineski SE, Collins TW, et al. Increasing research productivity in undergraduate research experiences: exploring predictors of collaborative faculty–student publications. CBE Life Sci Educ. 2017;16(3), Article ar42. doi: 10.1187/cbe.16-11-0326 28747352 PMC5589422

[21] Poncelet A, Collins S, Fiore D, et al. Identifying value factors in institutional leaders’ perspectives on investing in health professions educators. JAMA Netw Open. 2023;6(2):e2256193. doi: 10.1001/jamanetworkopen.2022.56193 36795413 PMC9936339

[22] Sanders M, Fiscella K. Underfunding for research training and career development: the impact on family medicine research. Fam Med. 2024;56(5):317–320. doi: 10.22454/FamMed.2024.453278 38506701 PMC11216765

[23] Diamond SJ, Thomas CR, Desai S, et al. Gender differences in publication productivity, academic rank, and career duration among U.S. academic gastroenterology faculty. Academic Med. 2016;91(8):1158–1163. doi: 10.1097/ACM.0000000000001219 27144993

[24] Holliday EB, Jagsi R, Wilson LD, et al. Gender differences in publication productivity, academic position, career duration, and funding among U.S. academic radiation oncology faculty. Academic Med. 2014;89(5):767–773. doi: 10.1097/ACM.0000000000000229 PMC4378901 24667510

[25] Zhou JZ, Lemelman BT, Done N, et al. Social media and the dissemination of research: insights from the most widely circulated articles in plastic surgery. Plast Reconstr Surg. 2018;142(2):555–561. doi: 10.1097/PRS.0000000000004598 30045187

[26] Porche DJ. President’s message—maintaining a program of research: deep work. Res Nurs Health. 2019;42(3):161–164. doi: 10.1002/nur.21945 30980417

[27] Horvath Z, Wilder RS, Guthmiller JM. The power of coaching: developing leaders and beyond. J Dent Educ. 2024;88(Suppl. 1):671–677. doi: 10.1002/jdd.13535 38758037

